# Soluble Guanylate Cyclase Stimulation Prevents Fibrotic Tissue Remodeling and Improves Survival in Salt-Sensitive Dahl Rats

**DOI:** 10.1371/journal.pone.0021853

**Published:** 2011-07-18

**Authors:** Sandra Geschka, Axel Kretschmer, Yuliya Sharkovska, Oleg V. Evgenov, Bettina Lawrenz, Andreas Hucke, Berthold Hocher, Johannes-Peter Stasch

**Affiliations:** 1 Cardiology Research, Bayer HealthCare, Wuppertal, Germany; 2 Global Biomarker, Bayer HealthCare, Wuppertal, Germany; 3 Institute of Nutritional Science, University of Potsdam, Potsdam, Germany; 4 Center for Cardiovascular Research, Institute of Pharmacology and Toxicology, Charité, Berlin, Germany; 5 Department of Anesthesia, Critical Care and Pain Medicine, Massachusetts General Hospital, Harvard Medical School, Boston, Massachusetts, United States of America; 6 Pathology, Bayer HealthCare, Wuppertal, Germany; 7 Institute of Pharmacy, Martin Luther University, Halle, Germany; Biological Research Center of the Hungarian Academy of Sciences, Hungary

## Abstract

**Background:**

A direct pharmacological stimulation of soluble guanylate cyclase (sGC) is an emerging therapeutic approach to the management of various cardiovascular disorders associated with endothelial dysfunction. Novel sGC stimulators, including riociguat (BAY 63-2521), have a dual mode of action: They sensitize sGC to endogenously produced nitric oxide (NO) and also directly stimulate sGC independently of NO. Little is known about their effects on tissue remodeling and degeneration and survival in experimental malignant hypertension.

**Methods and Results:**

Mortality, hemodynamics and biomarkers of tissue remodeling and degeneration were assessed in Dahl salt-sensitive rats maintained on a high salt diet and treated with riociguat (3 or 10 mg/kg/d) for 14 weeks. Riociguat markedly attenuated systemic hypertension, improved systolic heart function and increased survival from 33% to 85%. Histological examination of the heart and kidneys revealed that riociguat significantly ameliorated fibrotic tissue remodeling and degeneration. Correspondingly, mRNA expression of the pro-fibrotic biomarkers osteopontin (OPN), tissue inhibitor of matrix metalloproteinase-1 (TIMP-1) and plasminogen activator inhibitor-1 (PAI-1) in the myocardium and the renal cortex was attenuated by riociguat. In addition, riociguat reduced plasma and urinary levels of OPN, TIMP-1, and PAI-1.

**Conclusions:**

Stimulation of sGC by riociguat markedly improves survival and attenuates systemic hypertension and systolic dysfunction, as well as fibrotic tissue remodeling in the myocardium and the renal cortex in a rodent model of pressure and volume overload. These findings suggest a therapeutic potential of sGC stimulators in diseases associated with impaired cardiovascular and renal functions.

## Introduction

Tissue remodeling and degeneration ultimately resulting in a loss of function are observed in various organs including the heart, kidneys and vasculature in malignant arterial hypertension. Osteopontin (OPN), plasminogen activator inhibitor-1 (PAI-1) and tissue inhibitor of matrix metalloproteinase-1 (TIMP-1) are among the pro-fibrotic mediators that have been attributed to tissue degeneration and progression of cardiovascular disease. Preclinical and clinical evidence indicates that increased levels of these mediators are associated with a loss of function of several target organs including the heart and kidneys [1], [2]. Osteopontin, also called secreted phosphoprotein-1 or uropontin, is a cytokine-like pro-fibrotic mediator that stimulates extracellular matrix formation thus contributing to tissue remodeling [3]. Increased production of OPN has recently been shown to correlate with the severity of chronic heart failure and pulmonary hypertension (PH), and it is an independent predictor of death [4], [5]. In addition, OPN is attributed to the loss of renal function in patients with chronic kidney disease [6]. Likewise, an up-regulation of PAI-1 expression in the myocardium and vasculature is associated with increased accumulation of extracellular matrix and progression of cardiovascular disease [7]. Elevated plasma levels of PAI-1 also predict cardiovascular events and cardiovascular mortality in dialysis patients [8], [9]. Similarly, increased circulating TIMP-1 levels are associated with end-organ tissue remodeling and progression of cardiovascular and renal diseases [9], [10].

A novel pharmacological principle for treatment of different forms of cardiovascular disease and PH is based on the direct stimulation of the soluble guanylate cyclase (sGC) [11], [12], [13]. The present study was conducted to evaluate the effects of the new, selective and highly potent sGC stimulator riociguat on cardiac and renal tissue remodeling and organ function under conditions of chronic pressure and volume overload. Riociguat has a dual mode of action: it sensitizes sGC to endogenously produced nitric oxide (NO), while also directly stimulating sGC independently of NO [13]. Riociguat has been shown to improve hemodynamics and provide end-organ protection in several experimental models of PH and renal failure [14], [15]. The drug is currently undergoing phase III clinical trials in patients with pulmonary arterial hypertension and chronic thromboembolic pulmonary hypertension, having shown encouraging results in non-randomized studies.

## Results

### Survival rate, body weight, and hemodynamics

**Figure 1:**
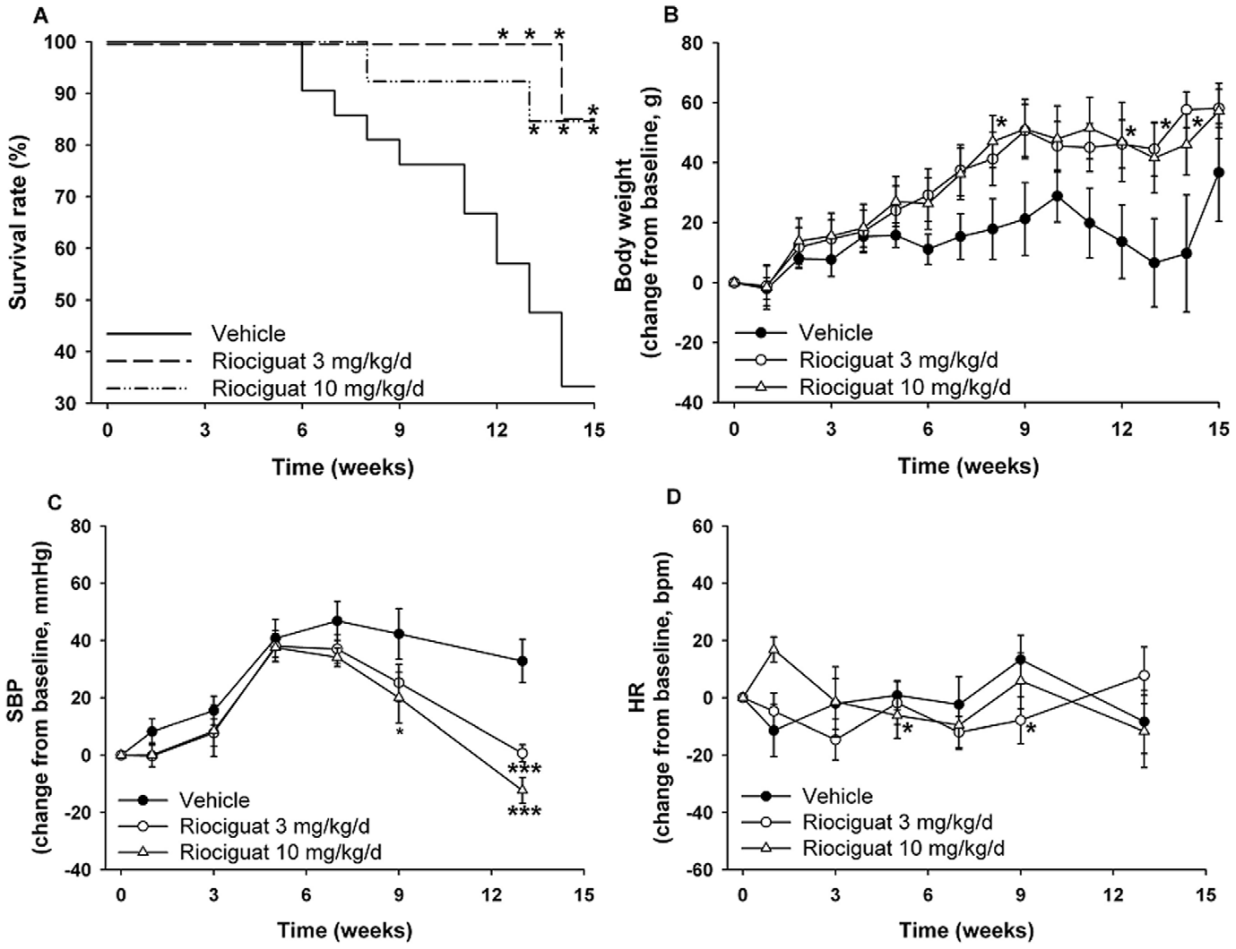
Effects of riociguat on survival (A), body weight (B), systolic blood pressure (C) and heart rate (D) in Dahl/ss rats maintained on a high-salt diet. Vehicle- (n = 21) and riociguat-treated (3 or 10 mg/kg/d, n = 13 per group) rats were observed for 14 weeks. Data are mean±SEM; *p<0.05, ***p<0.001 vs. the vehicle-treated animals.

The mortality of salt-loaded rats was significantly decreased in the riociguat-treated groups compared to the animals receiving vehicle alone. Only seven out of 21 (33%) vehicle-treated animals survived until the end of the study. In both of the riociguat-treated groups only two out of 13 rats died during the course of the study (85% survival) (p<0.05 vs. the vehicle group; Figure 1A). During the observation period the body weight of the vehicle group increased by 36.7±16.3 g, whereas the body weight of animals treated with riociguat 3 or 10 mg/kg/d increased by 58.1±6.4 g and 57.2±9.2 g, respectively (Figure 1B). Systolic blood pressure and heart rate were measured before starting the treatments and at several time points during the study, as depicted in the Figures 1C and 1D. During the first six weeks systolic blood pressure increased in all groups. In the vehicle-treated animals a maximum increase in systolic blood pressure was noted by week 7, and it was 32.8±7.6 mmHg above the baseline at the end of the study. In contrast, systolic blood pressure returned to the baseline in the animals treated with riociguat 3 mg/kg/d (0.7±3.0 mmHg) and was slightly reduced by riociguat 10 mg/kg/d (−12.3±4.5 mmHg). No significant changes in heart rate were observed.The body weight and relative organ weights (related to body weight) are presented in Table 1. The relative weights of the hearts, the left ventricles, and both kidneys were significantly lower in the animals treated with riociguat 10 mg/kg/d when compared to the vehicle group.

**Table 1:** Organ weights in the vehicle- and riociguat-treated Dahl/ss rats maintained on a high-salt diet.

	Vehicle (n = 17)	Riociguat (3 mg/kg/d) (n = 13)	Riociguat (10 mg/kg/d) (n = 13)
Body weight (g)	385±16	401±6	401±9
Relative heart weight (g/kg)	4.4±0.3	4.1±0.3	3.7±0.1**
Relative left ventricle weight (g/kg)	3.3±0.2	3.0±0.2	2.7±0.1*
Relative right ventricle weight (g/kg)	0.6±0.02	0.7±0.09	0.6±0.01
Relative left kidney weight (g/kg)	5.1±0.3	4.5±0.1**	4.2±0.1***
Relative right kidney weight (g/kg)	5.3±0.3	4.4±0.1**	4.2±0.08***

Measurements were obtained at the study end. Data are mean±SEM;

*p<0.05,

**p<0.01,

***p<0.001 vs. the vehicle-treated animals.

### Cardiac echocardiographic measurements

The results of cardiac echocardiographic measurements are summarized in Table 2. Overall, riociguat significantly improved various echocardiographic parameters as compared to the vehicle-treated controls. Fractional shortening was markedly reduced in the vehicle group, whereas it was maintained within the normal range by riociguat (p<0.05). While the relative increases in thickness of the intraventricular septum and the left free wall nearly doubled, the left free wall to intraventricular septum ratio and the ventricular diameters in diastole and systole were significantly reduced by riociguat (p<0.05).

**Table 2:** Cardiac echocardiographic measurements in the vehicle- and riociguat-treated Dahl/ss rats maintained on a high-salt diet.

	Vehicle (n = 17)	Riociguat (3 mg/kg/d) (n = 13)	Riociguat (10 mg/kg/d) (n = 13)
Fractional shortening (%)	19.7±1.8	43.9±1.8***	46.5±2.2***
Relative increase in thickness of IVS (%)	28.2±5.5	53.9±5.6**	54.5±4.4**
Relative increase in thickness of LfW (%)	29.0±7.1	45.8±6.1*	61.6±6.0**
LfW∶IVS	1.4±0.1	1.2±0.1*	1.0±0.03***
LfW diastole (mm)	2.3±0.1	2.6±0.1*	2.2±0.1
LVD diastole (mm)	8.9±0.2	7.7±0.3**	7.9±0.1**
IVS diastole (mm)	1.7±0.1	2.3±0.1***	2.2±0.1***
LVD systole (mm)	7.2±0.2	4.4±0.3***	4.2±0.2***

IVS = intraventricular septum, LfW = left free wall, LVD = left ventricular diameter in diastole or systole. Measurements were obtained at the study end. Data are mean±SEM;

*p<0.05,

**p<0.01,

***p<0.001 vs. the vehicle-treated animals.

### Plasma and urinary biochemical markers

Blood samples were obtained at the end of the study (Table 3). No significant intergroup differences were observed in plasma concentrations of ALT, GLDH, LDH, CK, creatinine, urea, protein, albumin, plasma renin activity or angiotensin I. Plasma concentrations of uric acid, AST, and the natriuretic peptides ANP and BNP were significantly reduced by riociguat (p<0.05). Urine samples were taken before starting treatments and at the end of the study. There were no significant intergroup differences in renal function parameters at the beginning of the study (data not shown). At the termination of the study (Table 4), diuresis and urinary excretion of urea, protein, Na^+^ and K^+^ were significantly reduced by riociguat as compared to the vehicle-treated controls (p<0.05). In contrast, urinary excretion of cGMP was markedly increased in both riociguat-treated groups (p<0.05).

**Table 3:** Plasma biochemical measurements in the vehicle- and riociguat-treated Dahl/ss rats maintained on a high-salt diet.

	Vehicle (n = 17)	Riociguat (3 mg/kg/d) (n = 13)	Riociguat (10 mg/kg/d) (n = 13)
AST (U/L)	109±18	73±4*	80±7*
ALT (U/L)	77±9	66±3	63±4
GLDH (U/L)	24±7	14±2	16±3
LDH (U/L)	523±123	505±50	398±56
CK (U/L)	282±48	310±22	250±30
Creatinine (µmol/l)	51±3	48±1	50±1
Urea (mmol/l)	7.4±1.0	7.8±0.5	9.2±0.4
Protein (g/l)	63±1	62±0.4	62±0.7
Uric acid (µmol/L)	53±8	24±2***	28±3**
Albumin (g/L)	27±0.7	28±0.5	29±0.7
PRA (ng/ml/h)	1.5±0.3	1.7±0.4	1.3±0.3
Angiotensin I (ng/ml)	0.6±0.1	0.6±0.1	0.5±0.1
ANP (pg/ml)	121±45	36±16**	20±5**
BNP (pg/ml)	16±4	7.9±2.6*	6.2±1.0*

AST = aspartate amino transferase, ALT = alanine amino transferase, GLDH = glutamate dehydrogenase, LDH = lactate dehydrogenase, CK = creatinine kinase, PRA = plasma renin activity, ANP = atrial natriuretic peptide, BNP = B-type natriuretic peptide. Data are mean±SEM;

*p<0.05,

**p<0.01,

***p<0.001 vs. the vehicle-treated animals.

**Table 4:** Parameters of renal function in the vehicle- and riociguat-treated Dahl/ss rats maintained on a high-salt diet.

	Vehicle (n = 17)	Riociguat (3 mg/kg/d) (n = 13)	Riociguat (10 mg/kg/d) (n = 13)
Diuresis (ml/kg/h)	4.7±0.7	1.6±0.2***	1.5±0.2***
Creatinine (µmol/kg/h)	9.6±0.8	11±0.8	11±0.8
Urea (µmol/kg/h)	787±136	243±32***	207±48***
Protein (mg/kg/h)	31±4.8	18±2.6**	9.5±2.0***
Na^+^ (µmol/kg/h)	910±199	282±50**	259±59**
K^+^ (µmol/kg/h)	230±34	147±13*	149±19*
cGMP (pmol/h)	880±145	3317±355***	4151±491***

Measurements were obtained at the study end (collection period of 8 hrs). Data are mean±SEM;

*p<0.05,

**p<0.01,

***p<0.001 vs. the vehicle-treated animals.

### mRNA expression of pro-fibrotic biomarkers

**Figure 2:**
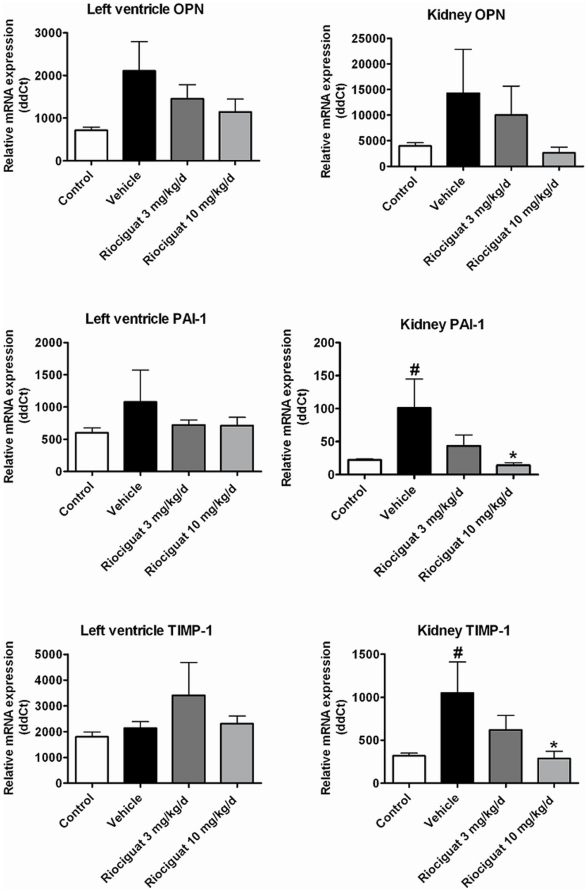
Effects of riociguat on mRNA expression of osteopontin (OPN), plasminogen activator inhibitor-1 (PAI-1) and tissue inhibitor of matrix metalloproteinase-1 (TIMP-1) in the left ventricle and the renal cortex in the vehicle (n = 7) - and riociguat-treated (3 or 10 mg/kg/d, n = 11 per group) Dahl/ss rats maintained on a high-salt diet. Healthy, age-matched animals were used as controls (n = 10). Data are mean±SEM; *p<0.05 vs. the vehicle-treated animals; #p<0.05 vs. healthy controls.

The gene expression of the pro-fibrotic biomarkers OPN, TIMP-1 and PAI-1 were determined in the hearts and kidneys of the pressure- and volume-overloaded Dahl/ss rats (Figure 2). Compared to healthy control animals, the relative increase in mRNA expression of the pro-fibrotic biomarkers was detected in all tissue samples in the vehicle group. In particular, mRNA expression of PAI-1 and TIMP-1 was significantly increased in the renal cortex (p<0.05). Treatment with riociguat prevented the mRNA up-regulation of OPN, TIMP-1, and PAI-1 in the renal cortex (p<0.05). Riociguat also attenuated mRNA expression of OPN, TIMP-1, and PAI-1 in the left ventricle.

### Protein expression of pro-fibrotic biomarkers

**Figure 3:**
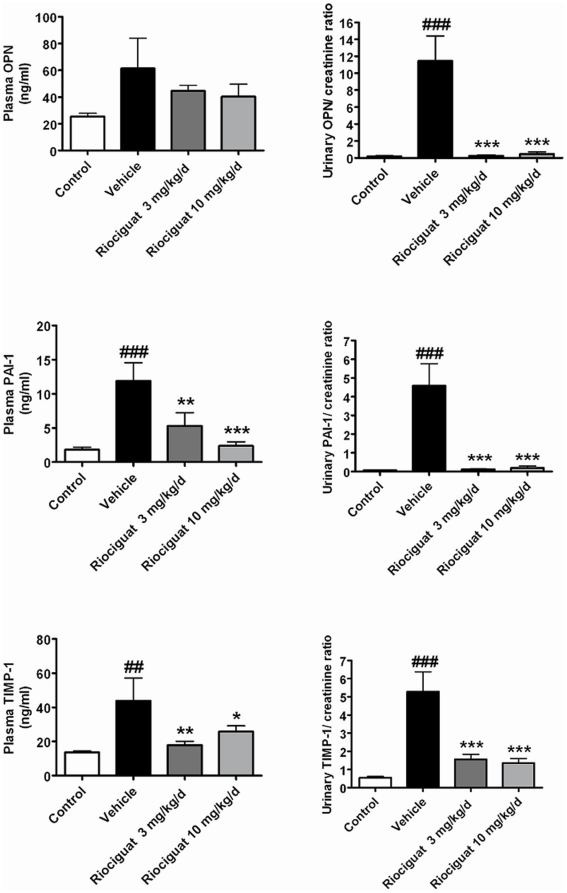
Effects of riociguat on plasma and urinary protein levels of osteopontin (OPN), plasminogen activator inhibitor-1 (PAI-1) and tissue inhibitor of matrix metalloproteinase-1 (TIMP-1) in the vehicle (n = 7) - and riociguat-treated (3 or 10 mg/kg/d, n = 11 per group) Dahl/ss rats maintained on a high-salt diet. Healthy, age-matched animals were used as controls (n = 10). Data are mean±SEM; *p<0.05, **p<0.01, ***p<0.001 vs. the vehicle-treated animals; ##p<0.01, ###p<0.001 vs. healthy controls.

Protein expression of OPN, TIMP-1, PAI-1 in plasma and urine was markedly elevated following salt loading (Figure 3). This increase in protein expression of the pro-fibrotic biomarkers was significantly reduced by riociguat (p<0.05).

### Histopathology

Histopathological evaluation of the kidneys and the hearts of the vehicle- and riociguat-treated rats was performed in order to elucidate effects of riociguat on tissue fibrosis (Table 5). The vehicle-treated animals demonstrated a profound tubulopathy in the renal cortex and extensive myocardial fibrosis in the subendocardial regions of the left ventricle. Treatment with riociguat attenuated degenerative and inflammatory changes in the kidneys and the heart. Histopathological examination of the kidneys revealed that riociguat significantly reduced glomerulosclerosis and interstitial and perivascular fibrosis compared to the vehicle-treated rats. Likewise, cardiac histopathology revealed that interstitial fibrosis and myocyte diameter were significantly decreased in the riociguat-treated animals compared to the vehicle group. No significant changes were found in the degree of cardiac perivascular fibrosis.

**Table 5:** Histopathological evaluation of the hearts and kidneys from the vehicle- and riociguat-treated Dahl/ss rats maintained on a high-salt diet.

	Vehicle (n = 17)	Riociguat (3 mg/kg/d) (n = 13)	Riociguat (10 mg/kg/d) (n = 13)
Glomerulosclerosis (score)	3.03±0.39	2.63±0.06**	2.56±0.04***
Renal interstitial fibrosis (%)	3.04±0.08	2.50±0.08***	2.14±0.09***
Renal perivascular fibrosis (%)	2.92±0.04	2.34±0.06***	2.20±0.04***
Myocyte diameter (µm)	11.20±1.3	9.99±0.93**	9.14±0.69***
Cardiac interstitial fibrosis (%)	1.93±0.31	0.82±0.14**	0.95±0.16*
Cardiac perivascular fibrosis (%)	2.57±0.18	2.33±0.10	2.55±1.00

Data are mean±SEM;

*p<0.05,

**p<0.01,

***p<0.001 vs. the vehicle-treated animals.

## Discussion

The recent discovery of sGC stimulators including riociguat (BAY 63-2521) offers an attractive option for the management of cardiovascular disease associated with underlying endothelial dysfunction. This novel therapeutic approach is based on a molecular mechanism that is fundamentally different from established vascular targets [12], [16]. Riociguat has a dual mode of action: it sensitizes sGC to endogenously produced NO and can also increase sGC activity in the absence of NO, causing vasorelaxation and anti-proliferative and anti-fibrotic effects. This dual mode of action is biologically important because the NO levels are decreased in various cardiopulmonary diseases [11], [12]. Importantly, in addition to its regulatory effects on vascular tone, NO may also exert numerous cytotoxic effects, largely attributed to the reactive oxidant peroxynitrite, which is formed from the diffusion-controlled reaction of NO with superoxide. Peroxynitrite interacts with lipids and proteins, altering cellular signaling, disrupting mitochondrial function, and damaging DNA, which can eventually lead to cellular dysfunction and death [17]. Hence, the novel pharmacological strategy of direct sGC stimulation may circumvent potentially detrimental effects of NO by acting further downstream in the NO-sGC-cGMP signaling cascade.

Recent studies have demonstrated that riociguat improves pulmonary hemodynamics and prevents or even reverses structural remodeling including the right ventricular hypertrophy and muscularisation of small pulmonary arteries in two experimental models of PH, namely hypoxia-induced PH in mice and monocrotaline-induced PH in rats [14]. Riociguat is currently undergoing phase III clinical trials in patients with pulmonary arterial hypertension and chronic thromboembolic PH. Clinical phase IIb studies showed that riociguat is well tolerated, easily administered and produces significant and long-lasting improvements in pulmonary hemodynamics, exercise capacity and functional class in patients with PH [13], [18], [19], [20].

The vascular effects of riociguat are not limited to the pulmonary vasculature [18]. We have very recently demonstrated, for the first time, that riociguat provides marked protection against cardiac and renal end-organ damage in experimental low-renin and high-renin models of hypertension [15]. To further investigate the therapeutic potential of riociguat, the present study was designed with a focus on the cardio-renal consequences of a long-term treatment with riociguat, including assessment of hemodynamics, cardiac and renal morphology, cardiovascular hormones, and mortality in Dahl/ss rats exposed to a high salt intake. Moreover, we measured protein expression of pro-fibrotic biomarkers (OPN, TIMP1 and PAI-1) to better characterize the anti-fibrotic effects of riociguat.

Inbred Dahl/ss rats maintained on a high salt diet (8% NaCl) are commonly used as a clinically relevant animal model to investigate salt-sensitive hypertension and cardiac and renal end-organ damage [21], [22]. In our study, these animals developed arterial hypertension and cardiac and renal hypertrophy and remodeling with impaired systolic and renal functions. Morphologically, cardiac and renal damage was characterized by vascular lesions, interstitial fibrosis and glomerulosclerosis. In response to riociguat, we found a significant attenuation of cardiac and renal hypertrophy and fibrosis and improved renal function in the Dahl/ss rats, as compared to the vehicle-treated animals. These effects were accompanied by a corresponding improvement of systolic cardiac function including fractional shortening. Importantly, sGC stimulation by riociguat resulted in a reduced mortality in the Dahl/ss rats exposed to a high salt diet. Our findings highlight the clinical relevance of the structural improvements induced by riociguat and provide further evidence that sGC stimulators might be utilized as a new therapeutic option for management of arterial hypertension and heart failure.

Increased oxidative stress, associated with an imbalance between pro-oxidants and antioxidants, is a risk factor for developing various cardiac and renal diseases [23], [24]. Essential hypertension in humans and salt-sensitive hypertension in animal models are associated with progressive renal damage and increased formation of reactive oxygen species [23], [25]. Manning et al. [23] have reported an increased renal superoxide production in Dahl/ss rats maintained on a high salt diet due to decreased renal levels of antioxidants including superoxide (SOD). Furthermore, the same research group [24] has demonstrated that treatment of Dahl/ss rats maintained on a high salt diet with the SOD mimetic tempol decreased mean arterial blood pressure and attenuated renal dysfunction. Moreover, St Lezin et al. [26] have shown that urinary cGMP excretion is impaired in Dahl/ss rats compared to control Dahl/sr rats. Whether this decrease in cGMP production is due to decreased bioavailability of L-arginine (the substrate for NO production), down-regulation or uncoupling of the endothelial NO synthase (eNOS), inactivation of NO by superoxide anion, increased plasma concentrations of the endogenous eNOS inhibitor asymmetric dimethylarginine, downregulation of sGC and/or alteration of the redox state of sGC through oxidative stress or a combination of all of the above is still unclear. Chen and Sanders [27] have also demonstrated that NO production is affected in Dahl/ss rats exposed to a high salt diet. Treatment with L-arginine decreased blood pressure and increased urinary cGMP excretion as a result of increased NO production. However, the direct stimulation of sGC is likely to offer a more advantageous approach in the setting of impaired NO production.

Although sGC agonists selectively stimulate the native sGC containing the reduced heme-moiety [12], [16], increased cGMP levels in urine in the present study indicate that riociguat might also stimulate sGC under conditions of oxidative stress and sensitize sGC to endogenously produced NO. Our results are in agreement with the recent findings that the sGC stimulator BAY 41-2272 (a close chemical analogue of riociguat) increases renal cGMP production and thereby improves renal NO-cGMP signaling, and prevents progression of the anti-Thy-1-induced chronic renal fibrosis [28]. Thus, the pharmacological enhancement of renal cGMP levels by sGC stimulation might provide protection against renal injury.

Histopathological analysis revealed strong anti-remodeling effects of riociguat as demonstrated by decreased tissue fibrosis and hypertrophy in the heart and kidneys. It is important to mention that these structural changes were also observed in the low-dose riociguat group. Masuyama et al. [29] tested the therapeutic potential of stimulating sGC with BAY 41-2272 administered for 14 days at a low dose (which had no effect on blood pressure) in a rat model of the angiotensin II-induced hypertension and cardiac remodeling. BAY 41-2272 prevented the angiotensin II-induced cardiac remodeling and fibrosis via a local, cGMP-dependent mechanism. In our study, a marked reduction of myocyte diameter was observed in the Dahl/ss rats treated with riociguat as compared with the animals receiving vehicle alone. In addition, riociguat attenuated interstitial fibrosis and glomerulosclerosis. These effects of riociguat in the salt-loaded Dahl/ss rats are consistent with previous studies in the L-NAME-treated renin transgenic rats and the 5/6 nephrectomized rats, where riociguat showed potent protection against cardiac and renal organ damage [15]. In agreement with these findings, plasma and urine protein levels of the pro-fibrotic biomarkers OPN, TIMP-1 and PAI-1 were elevated in the vehicle group and markedly decreased by riociguat, ultimately resulting in preservation of cardiac and renal functions. Masuyama et al. [29] have also showed that BAY 41-2272 downregulates collagen I and transforming growth factor mRNA levels in the left ventricle in a rat model of the angiotensin II-induced hypertension. Moreover, Peters et al. [28] have reported a decrease in collagen accumulation, protein and mRNA expression of TGF-β1, fibronectin, and PAI-1 by BAY 41-2272 in a model of progressive anti-Thy1-induced chronic glomerulosclerosis. Thus, the pro-fibrotic biomakers measured in our and other studies could be used to monitor clinical efficacy of this new class of drugs.

The relationship between the NO-sGC-cGMP pathway and profibrotic biomarker expression remains to be elucidated. Johnson et al. [30] have observed that OPN expression in Dahl rats strongly correlates with the degree of hypertension and, furthermore, that OPN is a potent inhibitor of inducible NO synthase expression. In patients, circulating levels of OPN, PAI-1, and TIMP-1 increase with the progression of chronic heart failure and PH [4], [5]. In addition, OPN expression coincides with tissue degeneration in the myocardium and coronary arteries, and its cytokine-like mode of action promotes tissue remodeling via binding to integrins and modulation of extracellular matrix formation [3], [4]. Similarly, an increased expression PAI-1 and TIMP-1 that contributes to extracellular matrix deposition has been observed in the hearts and kidneys of patients with chronic heart and kidney failure [2], [7]–[10], [1], [31]. A direct molecular mechanism linking sGC stimulation and regulation of the expression of these genes has not been identified yet. Importantly, the sGC stimulator riociguat markedly reduced the expression of OPN, PAI-1 and TIMP-1 in the present model of chronic volume- and pressure-overload in Dahl/ss rats.

There are some limitations of our study. First, the histopathological analysis revealed marked kidney damage but the renal function was not as much affected as expected. Secondly, healthy control rats could have been included for baseline hemodynamic measurements and analyses of plasma and urinary biochemical parameters. However, several previous studies have also demonstrated stable baseline data in healthy animals, as well as impaired renal function and concomitant tissue remodeling in the heart and kidneys in the Dahl/ss rats maintained on a high-salt diet [22], [32], [33], [34].

In conclusion, the present study demonstrates that the sGC stimulator riociguat attenuates systemic hypertension and cardiac and renal fibrotic tissue degeneration, improves systolic heart function and markedly increases survival in a model of chronic volume- and pressure-overload in Dahl/ss rats. These protective effects are accompanied by decreased expression of the pro-fibrotic biomarkers OPN, TIMP-1 and PAI-1, which have been shown to correlate with the severity of cardiovascular diseases and end-organ damage in clinical studies. Further studies are needed to clarify the relationship between these biomarkers and the NO-sGC-cGMP pathway. Our study provides an important step towards the clinical evaluation of the cardio-renal protective properties of sGC stimulators.

## Materials and Methods

### Chemicals

The sGC stimulator riociguat (BAY 63-2521; methyl-4,6-diamino-2-[1-(2-fluorobenzyl)-1H-pyrazolo[3,4-b]pyridin-3-yl]pyrimidin-5-ylmethylcarbamate) was synthesized as previously described [11].

### Animal model and treatments

This investigation conforms to the national Guide for the Care and Use of Laboratory Animals (Deutsches Tierschutzgesetz, May 18^th^, 2006) and EU directives (86/609) and was approved by a regional authority (Bezirksregierung Düsseldorf, Germany, Permit No. 50.05-240-26/05, No. 400/A14, approval date: March 21, 2005). Fifty seven 8-weeks old male Dahl salt sensitive (Dahl/ss) rats (Charles River Laboratories, Sulzfeld, Germany) were studied. Forty seven animals were fed a high-salt diet containing 8% NaCl (w/w) (Commercial diet Sniff, Soest, Germany) and randomly assigned to three treatment groups: vehicle alone (n = 21), riociguat 3 mg/kg/d (n = 13), and riociguat 10 mg/kg/d (n = 13). A separate age-matched control group (n = 10) was fed a standard diet without NaCl supplementation and used for baseline mRNA and protein quantification of the pro-fibrotic biomarkers. Riociguat was administrated once a day for 14 weeks by oral gavage in a suspension composed of Transcutol (10%), Cremophor (20%), and water (70%) in a volume of 2 ml/kg. The vehicle and control groups received only the Transcutol/Cremophor suspension.

Systolic blood pressure and heart rate were measured non-invasively via tail-cuff method (TSE Europe, Bad Homburg, Germany), and the body weight was recorded weekly. Individual urine samples were collected for the period of 8 hrs by diuresis cage collectors (Techiplast, Hohenpeissberg, Germany) on day 0 and at the end of the study.

At the study end, the animals were euthanized with intraperitoneal injection of pentobarbital, and blood samples were obtained. Hearts and kidneys were harvested, weighed and used for histological evaluation, and the tissue samples were taken for mRNA isolation and quantification of biomarker expression.

### Echocardiographic studies

High resolution transthoracic echocardiography (Sequia 512; Acuson, Mountain View, CA) was performed under isoflurane (2–2.5%) anesthesia at weeks 0 and 14. A 15-MHz linear array transducer was placed gently on the shaved right hemithorax. Parasternal left ventricular end-systolic and end-diastolic diameters and wall thickness were measured in the short axis view at the level of the papillary muscles using two dimensional guided M-mode imaging. The measurements were performed in accordance with the recommendations of the American Society of Echocardiography. Fractional shortening (FS) was caculated using the equation: FS (%) = ((LVDd-LVDs)/LVDd)*100, where LVDd is the left ventricular diameter in diastole and LVDs is the left ventricular diameter in systole [35]. All measurements were performed offline in DICOM-format by using SonoWin 4.1.6 (Meso, Mittweider, Germany).

### mRNA quantification of biomarkers by RTPCR

Tissue samples of the left ventricle myocardium or the renal cortex (approximately 2×2×2 mm) were cut off, immediately frozen on dry ice and stored at −80°C. Frozen tissue samples were ground in liquid nitrogen and extracted in 900 µl Trizol® (Invitrogen, Karlsruhe, Germany) according to the protocol provided by the manufacturer using glass bead homogenization in a FastPrep FP120 Bio-101 Savant vibration mill (MP Biochemicals, Eschwege, Germany). Subsequent to phenol-chloroform extraction and precipitation by isopropanol, DNA was eliminated from RNA samples by deoxyribonuclease I digest (Cat. No. 18068-015; Invitrogen, Karlsruhe, Germany). The ImProm-II TM Kit (Cat. No. A3800; Promega, Mannheim, Germany) was used for reverse transcription and first-strand cDNA synthesis. Real-time quantitative polymerase chain reactions (RTPCR) using the ABI Prism 7700 Sequence Detection System (Applied Biosystems, Carlsbad, CA) were performed to determine relative gene expression of OPN, TIMP-1, PAI-1. cDNA samples were amplified with a PCR mix containing Taq polymerase (qPCR MasterMix Plus, Ref. RT-QP2x-03-075+; Eurogentec, Seraing, Belgium) and primer sets with 6-FAM and TAMRA labeled probes (Operon Biotechnologies, Cologne, Germany) in 96-well or 384-well microtiter plates. 1–10 ng of cDNA samples were run in triplicates in reaction volumes of 25 µl (96-well MTP) or 20 µl (384-well MTP) under standard thermocycler conditions.

TaqMan probe sets were generated from mRNA sequence data (NCBI Genbank, U.S. National Library of Medicine, Bethesda, MD) using Primer Express Software v2.0 (Applied Biosystems). Relative gene expression was calculated using the delta Ct term (Applied Biosystems, User Bulletin No. 2) related to endogenous controls ribosomal protein L32 or beta-actin and Ct = 35 threshold value. The primer probe sets were generated from the following sequences (primers are listed for forward, reverse, and probe sequence, respectively):

NM_013226 ribosomal protein L32 (CTTACTGTGCTGAGATTGCTC, CCAGCTGTGCTGCTCTTTCA, CAATGTGTCCTCTAAGAACCGAAAAGCCAT), V01217 cytoplasmic beta-actin (ACCTTCAACACCCCAGCCA, CAGTGGTACGACCAGAGGCA, ACGTAGCCATCCAGGCTGTGTTGTCC), NM_009263 osteopontin (OPN, SPP1, AGCCATGACCACATGGACGA, GATTCGTCAGATTCATCCGAGT, AGACCATGCAGAGAGCGAGGA TTCTGTG), NM_008871 plasminogen activator inhibitor-1 (Serpine1, PAI-1, GGCCGACTTCACAAGTCTTTC, CGCCACTGTGCCGCTCTCGT, ACCAAGAGCAGCTCTCTGTAGCACA), NM_053819 tissue inhibitor of matrix metalloproteinase 1 (TIMP-1, CCGCAGCGAGGAGTTTCTC, GGCAGTGATGTGCAAATTTCC, TCGCGGGCCGTTTAAGGAA).

### Plasma and urinary analyses

Plasma creatinine, urea, creatinine kinase (CK), lactate dehydrogenase (LDH), glutamate dehydrogenase (GLDH), aspartate amino transferase (AST), alanine amino transferase (ALT), gamma-glutamyl tranferase (GGT), alkaline phosphatase (AP), total protein, and albumin were analyzed using a multiple channel analyzer (Synchron CX7; Beckmann, Hamburg) [36]. Atrial natriuretic peptide (ANP), angiotensin I, B-type natriuretic peptide (BNP), and plasma renin activity (PRA) were determined as described previously [37]. Plasma concentrations of osteopontin and TIMP-1 were quantified by enzyme linked immunosorbent assays (ELISA) according to the instructions of the manufacturer (R&D Systems, Minneapolis, MN), and total PAI-1 protein was measured by an ELISA kit from Loxo (Dossenheim, Germany). Urinary creatinine, urea, and total protein were analyzed on the Synchron CX7multiple channel analyzer [36]. Urine volume was measured gravimetrically and the excretion rates of sodium and potassium were determined using an electrolyte analyzer (Instrumentation Laboratory, Bedford, MA), as described elsewhere [38]. The urine cyclic guanosine monophosphate (cGMP) concentrations were measured using a commercially available radioimmunoassay kit (IBL, Hamburg, Germany). Individual urine samples from the rats were vortexed and centrifuged prior to biomarker quantification. The supernatants were diluted by an equal volume of phosphate buffered saline (PBS), and 50 µl of diluted urine was mixed with 50 µl of the sample buffer for detection of OPN, total PAI-1 protein and TIMP-1 using the same ELISA kits as for plasma analyses.

### Histopathological evaluation

Hearts and kidneys of the vehicle- and riociguat-treated animals were fixed in 10% neutral buffered formalin, embedded in Paraplast A and sectioned. Sections were stained with hematoxylin and eosin (H&E), Sirius Red, Periodic Acid-Schiff (PAS) and Elastica-van Gieson. Quantitative stereology was performed using a computer-aided image analysis system [39] and cardiac and renal morphology (interstitial fibrosis, perivascular fibrosis and glomerulosclerosis) were assessed as previously described [36], [40]. The severity of interstitial and perivascular fibrosis was evaluated following Sirius Red staining using computer-aided histomorphometry (Carl Zeiss Jenaoptik, Jena, Germany). PAS positive material within the glomeruli was defined as glomerulosclerosis. Perivascular fibrosis and glomerulosclerosis were assessed using a semi-quantitative score by two independent investigators blinded to the study groups. Microscopic pictures of kidney and heart sections following Elastica-van Gieson staining were generated for arterial blood vessel examination. The area contents of the media and the lumen of renal or myocardial arteries were analyzed using the ImageJ processing software developed at the National Institutes of Health (Bethesda, MD), and the media-to-lumen ratio was calculated serving as a marker for arterial wall thickening. Myocyte diameter was determined using H&E stained sections of the heart and subsequently evaluated by the ImageJ software.

### Statistical analysis

Data are presented as mean±SEM. The treatment effects were tested using a one-way ANOVA followed by a Newman-Keuls *post hoc* test, where appropriate (GraphPad Software 4.02, San Diego, CA). Survival analysis was performed using Kaplan-Meier analysis and the log-rank-test. A value of p<0.05 was considered statistically significant.
